# The effect of prophylactic rewarming on postoperative nausea and vomiting among patients undergoing laparoscopic hysterectomy: a prospective randomized clinical study

**DOI:** 10.1590/1516-3180.2020.0059.R2.06072020

**Published:** 2020-10-09

**Authors:** DongDong Liang, YuanLu Shan, Leilei Wang

**Affiliations:** I MD. Anesthesiologist, Department of Anesthesiology, The First Affiliated Hospital of Wenzhou Medical University, Ouhai Area, Wenzhou City, Zhejiang Province, China.; II MD. Anesthesiologist, Department of Anesthesiology, The First Affiliated Hospital of Wenzhou Medical University, Ouhai Area, Wenzhou City, Zhejiang Province, China.; III MD. Anesthesiologist, Department of Anesthesiology, The First Affiliated Hospital of Wenzhou Medical University, Ouhai Area, Wenzhou City, Zhejiang Province, China.

**Keywords:** Anesthesia, general, Hysterectomy, Postoperative nausea and vomiting, Temperature, Visual analogue scale, PONV, QoR-40, Recovery, Rewarming

## Abstract

**BACKGROUND:**

Postoperative nausea and vomiting (PONV) is a common complication from general anesthesia that impacts on postoperative recovery.

**OBJECTIVE:**

To evaluate prophylactic rewarming following general anesthesia, so as to decrease the incidence of PONV among patients undergoing laparoscopic hysterectomy.

**DESIGN AND SETTING:**

Prospective randomized clinical study at a hospital in China.

**METHODS:**

Sixty-two patients were randomly assigned into two groups. The forced air warming (FAW) group received pre-warmed Ringer's solution with FAW until the end of surgery. The control group received Ringer's solution without FAW. The pre-warmed Ringer's solution was stored in a cabinet set at 40 °C. The FAW tube was placed beside the patient's shoulder with a temperature of 43 °C.

**RESULTS:**

Sixty patients completed the study. The FAW group showed significant differences versus the controls regarding temperature. At 6, 24 and 48 hours postoperatively, the incidences of PONV were 53.3%, 6.7% and 3.3% in the FAW group versus 63.3%, 30% and 3.3% in the controls. VAS scores were significantly lower in the FAW group than in the controls at 24 hours (P= 0.035). Forty-item questionnaire total scores in the FAW group were significantly higher than in the controls. The physical independence and pain scores at 24 hours and emotional support and pain scores at 48 hours in the FAW group were higher than in the controls (P < 0.05). There was no difference in hemodynamics or demographics between the two groups (P > 0.05).

**CONCLUSIONS:**

Prophylactic rewarming relieved PONV and improved the quality of postoperative recovery.

**CHINESE CLINICAL TRIAL REGISTER (ChiCTR):**

ChiCTR-IOR-17012901.

## INTRODUCTION

Postoperative nausea and vomiting (PONV) after general anesthesia has high incidence (20%-80%). It is an uncomfortable complication that causes distress for patients.^1^ It occurs much more frequently among high-risk patients (60-70%), such as females, individuals who suffer from motion sickness, nonsmokers and individuals with a history of PONV.^2^ Laparoscopic surgery is prone to induce postoperative nausea and vomiting, which significantly increases patients’ discomfort, such that they hardly take in any nutritious food, which thus results in extending their length of hospital stay.^3^ Multiple antiemetic drugs have been applied in clinics, but the efficacy of such treatment comes with risks of adverse events such as excessive sedation,^4^ dizziness, dry mouth, dysphoria, mood changes,^5^ tachycardia and extrapyramidal signs.

Besides drug therapy, nondrug therapy also provides some help in preventing occurrences of PONV. Intraoperative skin surface rewarming is a common and rapid method that not only can prevent hypothermia but also can improve postoperative comfort.^6^^,^^7^ Rein et al.^8^ and Hamza et al.^9^ showed that perioperative temperature protection increased skin blood flow and heat transfer, and also lowered the requirement for analgesics and promoted higher quality of recovery.^10^ Reflective blankets,^11^ forced-air warmers and warm socks have all been used clinically to prevent shivering and maintain subjective thermal comfort postoperatively,^12^^,^^13^ thereby indirectly minimizing development of PONV.

The underlying mechanisms of PONV are complex and relate to the patient's psychological state. Watcha and White believed that vagal stimuli from the intestinal tract could activate the emetic center and trigger chemoreceptors, which would result in a series of reactions to the onset of nausea and vomiting.^4^ Some clinical trials have shown that oral administration of warm water for four hours postoperatively had the capacity to significantly decrease the first flatus expulsion, relieve gastrointestinal spasms and help peristalsis return at an early stage of recovery.^14^

Therefore, we hypothesized that thermal protection for patients would prevent PONV and provide better benefit in recovery. To test this hypothesis, we applied forced-air warmers combined with warm liquid to maintain temperature fluctuation perioperatively; a 100-mm visual analogue scale (VAS) to evaluate overall postoperative PONV; and a 40-item questionnaire (QoR-40) to measure the quality of recovery.

## OBJECTIVE

The aim of this study was to evaluate prophylactic rewarming following general anesthesia, to guard against postoperative nausea and vomiting among patients undergoing laparoscopic hysterectomy.

## METHODS

The present study was registered in the Chinese Clinical Trial Register with the code ChiCTR-IOR-17012901. This was a prospective randomized study in which 62 patients who were candidates for laparoscopic hysterectomy under general anesthesia at a hospital in China were enrolled between July 2017 and March 2018. In accordance with the requirements of the ethics committee for clinical research (number 2017-162), the patients were given explanations about the purpose of the study protocol and they gave their written consent to participate. The clinical trial consent and QoR-40 questionnaire were explained to the patients one day before surgery.

From the surgical list, we identified the patients who were eligible to become involved in the clinical trial. Patients who conformed to the inclusion criteria were allocated before the surgery either to the forced air warming (FAW) group or to the control group by means of numbers in identical sealed envelopes, according to a random number table that was created through a computer by an independent statistician. One of the anesthesiologists (WLL) made an evaluation and recorded the data after the participants had signed the consent form.

An independent nurse who was not involved in caring for these patients opened the envelopes before the operation and prepared the fluids and FAW. The FAW tube was placed beside the patient's shoulder with the temperature at 43 °C. Two of the anesthesiologists (LDD, SYL), who were unaware of the allocation group, performed the general anesthesia and all intraoperative data recording, and another investigator (WLL) was in charge of all postoperative assessments, while blinded to the group identity.

### Subjects

The inclusion criteria were that the subjects needed to present the following: American Society of Anesthesiologists (ASA) physical status I/II; aged 20 to 60 years; consent to their participation in the study until the end; scheduled to undergo laparoscopic hysterectomy. Written informed consent was obtained from all subjects. All of them answered the QoR-40 questionnaire independently.

Presentation of any of the following were deemed to be exclusion criteria: allergy; bronchial asthma; coronary heart disease; obesity-related diabetes mellitus; hypertension; BMI > 30 kg/m^2^; cardiac, hepatic or renal dysfunction; psychiatric disease; chronic pain; fever; history of alcohol or opioid abuse; intake of any nonsteroidal analgesics or antiepileptic drugs within 48 hours before surgery; or history of gastrointestinal disease (peptic ulcer, Crohn's disease or ulcerative colitis). Patients were withdrawn from the groups if their laparoscopy was converted to open surgery.

Sixty female patients aged 20 to 60 years who presented ASA physical status I or II and had been scheduled for primary gynecological laparoscopic surgery were randomly assigned to two groups. Patients in the FAW group received pre-warmed Ringer's solution that was stored in a heating cabinet set at 40 °C and was applied with forced air warming (FAW) that was switched on until the end of surgery. Patients in the control group received normal general anesthesia with normal Ringer's solution, i.e. FAW was switched off. To ensure that the surgery went smoothly, we set the patients’ intraoperative temperature to be not lower than 35 °C. In the event of lower temperatures occurring in the control group, our intention was to stop the trial and take protective measures.

Anesthesia was induced in all patients by means of propofol 2 (mg/kg) and sufentanil (0.3-0.5 μg/kg), and intubation was done using cisatracurium (2 mg/kg). Anesthesia was maintained by means of sevoflurane, propofol and remifentanil. The bispectral index (BIS) was monitored to maintain it at 45-55 in order to control the infusion speed of anesthetic drugs.

Mechanical ventilation was performed to maintain PetCO_2_ at 35-40 mmHg. Sufentanil (0.1 mg/kg per 30 minutes) was administered during the surgery to provide analgesia. Intravenous ondansetron (8 mg) was administered to prevent postoperative nausea and vomiting. When patients presented spontaneous breathing, consciousness was recovered by using neostigmine and atropine, and then the tracheal tube was extracted.

### Measurement

1. Postoperative nausea and vomiting were evaluated and measured by means of a 100-mm VAS at the postoperative time points of 6 hours, 24 hours and 48 hours. Additionally, we recorded any occurrences of nausea and vomiting in the ward, and the number of times of using antiemetic drugs.
2. Core temperature was recorded by using a temperature probe placed in the nasal cavity. We set 37.0 °C as the starting temperature in both groups. The changes in nasal temperature were recorded as follows: ΔT_0_ (ΔT_0_ = 37.0 °C – the intubation temperature); ΔT_30_ (ΔT_30_ = intubation temperature – temperature 30 minutes after intubation), ΔT_60_ (ΔT_60_ = intubation temperature – temperature 60 minutes after intubation), ΔT_90_ (ΔT_90_ = intubation temperature – temperature 90 minutes after intubation).
3. The validated Chinese version of the QoR-40 questionnaire was used at three times: preoperatively (T0), 24 hours postoperatively (T1) and 48 hours postoperatively (T2).^15^^,^^16^ QoR-40 contains five subscales: physical comfort (PC), emotional state (ES), physical independence (PI), patient support (PS) and pain (P). Each item is rated on a scale of 1-5, and therefore the total score can range from a minimum of 40 to a maximum of 200. The QoR-40 questionnaire was used to measure the patients’ physical condition after anesthesia.
4. Perioperative hemodynamics: heart rate and mean arterial pressure (MAP) were recorded at the times of the baseline, intubation and 10 minutes, 20 minutes, 30 minutes, 40 minutes, 50 minutes and 60 minutes after induction of anesthesia, and at the end of surgery.
5. Occurrences of shivering^17^ (at the end of surgery and in the early postoperative period up to one hour) were evaluated and recorded in two groups, as follows:
  - grade 1: no shivering
  - grade 2: mild shivering, with slight facial and cervical muscle contraction
  - grade 3: moderate shivering, consisting of obvious shivering of the head and neck, shoulders, and/or extremities
  - grade 4: severe shivering, consisting of obvious shaking all over the body
6. The patients’ demographic profiles in the two groups were recorded, including age, body mass index, intraoperative sufentanil and remifentanil consumption, liquid dosage, time of extubation and durations of anesthesia and the operation.

### Data analysis and statistics

The demographic profiles were analyzed by means of the independent-sample t test. The paired-sample t test was used to test for significant differences in ΔT between the two groups. The Wilcoxon test with the Mann-Whitney U test was used to analyze PONV scores and QoR-40 scores. Repeated-measurement analysis of variance (ANOVA) followed by the Huynh-Feld correction was used for analysis on MAP and heart rate. Occurrences of shivering were tested using the chi-square test with Fisher's exact test.

All values were presented as means ± standard deviation (SD). All the analyses were performed using the SPSS statistical software (SPSS Inc., Chicago, Illinois, USA). P-values < 0.05 were considered statistically significant.

## RESULTS

### 1. Postoperative nausea and vomiting:

At 6 hours after the operation, the incidences of PONV were 53.3% (16/30) in the FAW group and 63.3% (19/30) in the control group, within which the vomiting rates were 20% (6/30) in the FAW group and 23.3% (7/30) in the control group. However, there was no statistically significant in VAS scores (P = 0.258). At 24 hours after the operation, the incidences of PONV were 6.7% (2/30) in the FAW group and 30% (9/30) in the control group, within which the vomiting rates in the two groups were equal, at 3.3% (1/30). The VAS scores in the control group were significantly higher than those in the FAW group (P = 0.035). At 48 hours after the operation, the incidences of PONV in the two groups were equal at 3.3% (1/30), and none of the subjects presented vomiting. There was no significant difference in VAS scores at 48 hours after the operation between the two groups (P = 0.981; Table 1).

**Table 1:** Postoperative nausea and vomiting according to visual analogue scale scores in the two groups

Time	Visual analogue scale score		
	FAW group n = 30	Control group n = 30	Pα
6 hours	2.53 ± 2.75	3.47 ± 3.13	0.258
24 hours	0.47 ± 1.78	1.00 ± 1.64	0.035*
48 hours	0.07 ± 0.37	0.10 ± 0.55	0.981

6 hours, 6 hours after operation; 24 hours, 24 hours after operation; 48 hours, 48 hours after operation;

α obtained through the Wilcoxon test with Mann-Whitney U test; visual analogue scale scores at 24 hours after operation, FAW group (*P = 0.035) versus control group; FAW = forced air warming.

Additionally, the proportions of the patients who presented a need for use of antiemetic drugs to relieve PONV in the ward were 46.7% (14/30) in the FAW group and 56.7% (17/30) in the control group. Ondansetron (44.3%), promethazine (1.7%) and metoclopramide (6.7%) were administered to prevent and treat nausea and vomiting in the ward.

### 2. Core temperature:

**Figure 1:**
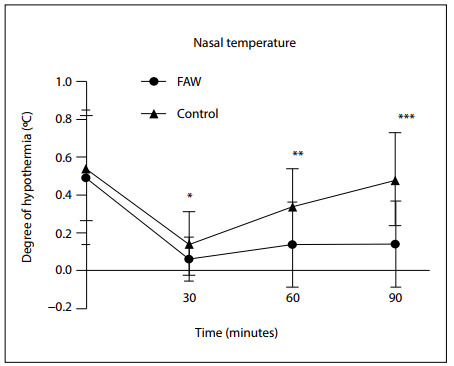
Changes to nasal temperature in the two groups. X axis encompasses the baseline of intubation and intubation after 30 minutes, 60 minutes and 90 minutes. Y axis represents the magnitude of the decline in temperature during the operation. All values are presented as means ± standard deviation (SD). Forced air warming (FAW) group versus control group at ΔT30 *P = 0.013, at ΔT60 **P = 0.001 and at ΔT90 ***P = 0.000, respectively.

Starting from the baseline of intubation, there was no difference in temperature drop between the two groups. At the time of 30 minutes after intubation, there was a statistical difference in the degree of temperature decline between the two groups (FAW: ΔT_30_ = 0.0467 ± 0.12243; control: ΔT_30_ = 0.1433 ± 0.16955; P = 0.013). At the time of 60 minutes after intubation, the degree of temperature decline in the FAW group was reduced. However, in the control group, the degree of temperature decline did not reduce, thus leading to a significant difference between the two groups (FAW: ΔT_60_ = 0.1367 ± 0.22664; control: ΔT_60_ = 0.3367 ± 0.20083; P = 0.001). At the time of 90 minutes after intubation, the degree of temperature decline in the FAW group was significantly reduced, compared with the control group (FAW: ΔT_90_ = 0.1400 ± 0.22834; control: ΔT_90_ = 0.3833 ± 0.24507; P = 0.000; Figure 1).

### 3. Results from QoR-40:

All the patients (n = 30 in each group) received the QoR-40 questionnaire at three times: before the operation (T0), 24 hours after the operation (T1) and 48 hours after the operation (T2). At T1, the patients in the control group had lower overall QoR-40 scores than the patients in the FAW group (P = 0.027) and lower scores for the PI and P dimensions (P = 0.032, P = 0.034 respectively). At T2, the overall QoR-40 scores in the two groups were higher and returning towards the preoperative level. Patients in the FAW group showed better recovery than those in the control group, with a statistically significant difference (P = 0.006). The ES and P dimensions in the control group had lower scores than those of the T group (P = 0.024 and P = 0.002, respectively; Table 2).

**Table 2:** QoR-40 scores at T0, T1 and T2 among the patients

		FAW group n = 30	Control group n = 30	Pα
**T0**				
	Overall	195.73 ± 5.41	196.67 ± 4.25	0.556
	Physical comfort (PC)	59.00 ± 1.53	58.97 ± 1.50	0.91
	Emotional state (ES)	43.07 ± 2.96	43.10 ± 3.53	0.658
	Physical independence (PI)	24.80 ± 0.76	24.87 ± 0.73	0.321
	Psychological support (PS)	34.80 ± 0.48	34.77 ± 0.43	0.573
	Pain (P)	34.10 ± 1.32	34.10 ± 1.185	0.877
**T1**				
	Overall	175.50 ± 9.63	170.47 ± 9.35	0.027*
	Physical comfort (PC)	50.77 ± 5.46	49.43 ± 4.75	0.233
	Emotional state (ES)	42.07 ± 3.48	41.27 ± 3.62	0.11
	Physical independence (PI)	17.27 ± 2.26	15.77 ± 2.53	0.032*
	Psychological support (PS)	34.57 ± 0.73	34.67 ± 0.48	0.857
	Pain (P)	30.90 ± 2.19	29.40 ± 2.92	0.034*
**T2**				
	Overall	190.20 ± 5.37	186.07 ± 6.50	0.006#
	Physical comfort (PC)	58.137 ± 2.21	57.70 ± 2.61	0.353
	Emotional state (ES)	43.80 ± 2.04	41.83 ± 6.09	0.024*
	Physical independence (PI)	20.20 ± 2.57	19.60 ± 2.76	0.18
	Psychological support (PS)	34.90 ± 0.31	34.90 ± 0.31	1
	Pain (P)	33.17 ± 1.56	31.33 ± 2.54	0.002#

Values are expressed as mean ± standard deviation (SD) or number of patients. T0, before surgery; T1, 24 hours after surgery; T2, 48 hours after surgery; FAW group, forced air warming group;

α obtained through the Wilcoxon test with Mann-Whitney U test;

* P < 0.05;

# P < 0.01. At T1 and T2, overall scores in FAW group (*P = 0.027 and ^#^P = 0.006, respectively) versus control group. At T1, PI and P scores in FAW group (*P = 0.032 and ^#^P = 0.034, respectively) versus control group. At T2, ES and P scores in FAW group (*P = 0.024 and ^#^P = 0.002, respectively) versus control group.

### 4. Perioperative hemodynamics:

**Figure 2:**
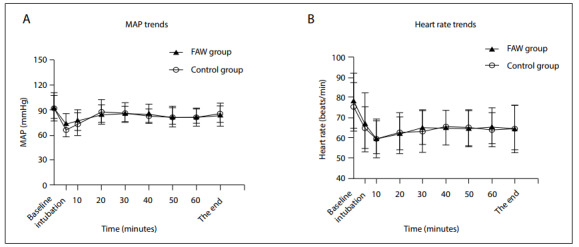
Perioperative hemodynamics. a. Mean arterial pressure (MAP) trends in the two groups. b. Heart rate trends in the two groups. Values are expressed as means ± standard deviation (SD). X axis encompasses the baseline intubation and 10 minutes, 20 minutes, 30 minutes, 40 minutes, 50 minutes and 60 minutes after induction of anesthesia. There were no significant differences between the two groups.

No significant differences were seen between the two groups in terms of the perioperative MAP and heart rate (HR) (FAW: MAP = 84.4000 ± 11.36555; control: MAP = 81.7233 ± 12.21111; P > 0.05; FAW: HR = 66.0844 ± 10.06888; control: HR = 64.9811 ± 9.96222; P > 0.05; Figure 2). Both MAP and heart rate values decreased at the time of tracheal cannulation and then maintained a lower level than the baseline. However, these values tended to remain within an acceptable range once surgery had commenced.

### 5. Occurrence of shivering:

Occurrences of shivering were associated with high incidence of low temperature, compared with the control group (P = 0.024; Table 3).

**Table 3:** Occurrences of shivering

Group	Occurrences of shivering	
	Yes (n = 30)	No (n = 30)
FAW group	0	30
Control group	6	24
Pα	0.024*	

Values are expressed as numbers of patients.

α obtained through the chi-square test + Fisher's exact test. Occurrences of shivering in the FAW group (*P < 0.05) versus control group. FAW, forced air warming.

### 6. Patient characteristics:

Sixty-two patients who were candidates for laparoscopic hysterectomy under general anesthesia were enrolled for this study. Two patients were excluded as a result of factors such as changes to the surgical procedure and blood sample loss. Thus, 60 female patients were included between July 2017 to March 2018, and were divided into two groups (FAW and control). There were no significant differences between the groups regarding age, body mass index, intraoperative sufentanil (34.80 ± 5.85 μg versus 35.53 ± 6.54 μg) and remifentanil consumption (679.00 ± 256.72 μg versus 728.27 ± 270.34 μg), liquid dosage (1033.33 ± 224.89 ml versus 1000.00 ± 227.43 ml), time of extubation, and durations of anesthesia and the operation (P > 0.05; Table 4).

**Table 4:** Demographic data of the patients included

Items	FAW group n = 30	Control group n = 30	P
Age; years	48.63 ± 4.41	47.17 ± 4.54	0.21
Body mass index; kg/m^2^	22.74 ± 2.66	23.64 ± 2.41	0.173
Duration of operation; minutes	77.30 ± 29.23	91.53 ± 31.91	0.077
Duration of anesthesia; minutes	116.87 ± 132.41	108.63 ± 31.197	0.741
Crystalloids; ml	1033.33 ± 224.89	1000.00 ± 227.43	0.57
Sufentanil; μg	34.80 ± 5.85	35.53 ± 6.54	0.65
Remifentanil; μg	679.00 ± 256.72	728.27 ± 270.34	0.472
Time of extubation, minutes	5.00 ± 3.09	5.83 ± 5.07	0.445

FAW group, forced air warming group; values are expressed as mean ± standard deviation (SD) or number of patients. No significant differences between the two groups.

## DISCUSSION

PONV is a commonly encountered symptom among patients in a variety of clinical settings.^18^ PONV causes distress for patients and affects postoperative recovery quality, although the precise mechanism is still unclear. The main finding in our study was that prophylactic rewarming (pre-warmed Ringer's solution with FAW) could effectively ameliorate the condition of PONV at 24 hours after the operation. It also helped to improve the quality of early recovery among these laparoscopic hysterectomy patients, 24 hours and 48 hours after the operation.

Perioperative hypothermia has been found to tend to induce occurrence of nausea and vomiting, in many studies.^19^^–^^21^ In our study, temperature values in both groups decreased markedly after intubation. However, the degree of temperature decline in the FAW group was reduced, compared with the control group, from the time of 30 minutes after intubation to the time of 90 minutes after intubation. The results suggested that pre-warming fluids applied in association with FAW were able to provide steady heat transfer throughout the surgical procedure and minimized the core temperature loss, which was caused mostly by surgical and anesthesia factors.

It is hard to maintain normothermia at a typical operating room temperature. Some studies have reported that general anesthesia has the capacity to reduce metabolic heat production by about 30%. However, perioperative warming devices may compensate for this.^22^

In our study, hypothermia possibly caused occurrences of PONV, notably at 24 hours after the operation (the rate of occurrence of nausea and vomiting was 6.7% in the FAW group versus 30% in the control group). VAS scores at 24 hours in the FAW group were much lower than those in the control group. This suggested that the patients in the FAW group were in a better physical condition at 24 hours after the operation, with low occurrence of PONV. However, the use of antiemetic drugs in the ward in the two groups was 46.7% in the FAW group and 56.7% in the control group.

Some studies have shown that occurrences of nausea are more resistant to interventions.^23^ The data from the ward suggested to us that clinicians in the ward were possibly prescribing antiemetic drugs as prophylaxis for PONV. Quigley et al. stated that most clinically encountered episodes of PONV were typically short-lived and self-limited.^24^ Because of the prophylactic antiemetic drugs, the number of times that patients in the FAW group asked for relief from nausea diminished.

In addition, we observed that frequency of occurrence of postoperative shivering increased in the control group. Along with PONV, shivering caused discomfort for the patients recovering from general anesthesia, even though none of them presented temperatures under 35 °C. This possibly implied that pre-warming decreased the risk of surgical complications. Patients were able to absorb nutrients earlier, which was conducive to recovery.^25^

Furthermore, the QoR-40 scores suggested that the higher these were, the faster and better the quality of recovery were. The FAW group showed better status for physical independence (PI) and pain (P) than the control group at 24 hours after the operation. Meanwhile, presence of pain itself increased the occurrences of PONV. Moreover, postoperative opioid administration likewise has been found to give rise to a high risk of PONV.^26^ At 48 hours after the operation, the ES scores in the FAW group were clearly higher than those in the control group.

Most patients in both groups lay in a semi-reclining position on the bed. Better body condition and peaceful psychological status would be expected to accelerate rehabilitation. However, we found that for some patients whose psychological status was poor at the outset, their condition could not be improved through surgery because their pessimism affected the functioning of their immune system.^27^^–^^29^

Some studies have demonstrated that the medial prefrontal cortex and the pregenual anterior cingulate cortex are involved in people's cognitive and emotion functioning. Vitaly Napadow showed that the presence of stress, emotion and fear conditioning was associated with increasing sensation of nausea in the brain through functional magnetic resonance imaging (fMRI).^30^ Some research has suggested that knowledge of the risk factors for nausea and vomiting, along with knowledge of health and affective factors, would lead to healthier behavior.^31^^,^^32^

At 24 hours and 48 hours after the operation, the total QoR-40 scores in the FAW group were significantly higher than those in the control group. The quality of recovery in the FAW group suggested that patients with pre-warming were not undergoing any intensely physiological stress reactions, such as PONV, shivering and heat loss.

There were some limitations to this study. Firstly, we did not test any serum biochemical parameters to reflect the patients’ inner reactions to nausea and vomiting through maintenance of normal temperature. Secondly, we did not test the PONV intensity scale, which could have provided supplementary data to explain the relationship between prophylactic rewarming and PONV.

## CONCLUSIONS

Prophylactic rewarming effectively relieved the condition of PONV and provided some help in improving the quality of postoperative recovery among these patients undergoing laparoscopic hysterectomy.
